# Synaptophysin, CD117, and GATA3 as a Diagnostic Immunohistochemical Panel for Small Cell Neuroendocrine Carcinoma of the Urinary Tract

**DOI:** 10.3390/cancers14102495

**Published:** 2022-05-19

**Authors:** Gi Hwan Kim, Yong Mee Cho, So-Woon Kim, Ja-Min Park, Sun Young Yoon, Gowun Jeong, Dong-Myung Shin, Hyein Ju, Se Un Jeong

**Affiliations:** 1 Asan Medical Center, Department of Pathology, University of Ulsan College of Medicine, 88, Olympic-ro 43 Gil, Songpa-gu, Seoul 05505, Korea; standupbau@hanmail.net (G.H.K.); yongcho@amc.seoul.kr (Y.M.C.); 2 Department of Pathology, Kyung Hee University Medical Center, Kyung Hee University College of Medicine, Seoul 02447, Korea; sowoonkim86@gmail.com; 3 Asan Medical Center, Asan Institute of Life Science, Seoul 05505, Korea; parkja09@naver.com (J.-M.P.); mysunyoung14@naver.com (S.Y.Y.); 4 AI Recommendation, T3K, SK Telecom, 65, Eulji-ro, Jung-gu, Seoul 04539, Korea; gowun.jeong@googlemail.com; 5 Asan Medical Center, Departments of Biomedical Sciences and Physiology, University of Ulsan College of Medicine, Seoul 05505, Korea; d0shin03@amc.seoul.kr (D.-M.S.); alal0903@naver.com (H.J.)

**Keywords:** carcinoma, neuroendocrine, urinary bladder, decision trees, immunohistochemistry, synaptophysin, negative results

## Abstract

**Simple Summary:**

While diagnosing a case of small cell neuroendocrine carcinoma (SCNEC) in the urinary tract, we found that the previous biopsy had been misdiagnosed as urothelial carcinoma (UC) because only chromogranin and synaptophysin were tested to define neuroendocrine differentiation and both tests were negative. This case led us to conduct this present study to define a panel of neuroendocrine markers to ensure the diagnosis of traditional neuroendocrine marker-negative SCNEC. We employed a decision tree classifier algorithm to analyze the expression of 17 immunohistochemical markers and found that the extent of synaptophysin (>5%) and CD117 (>20%) and the intensity of GATA3 (negative or weak) are major parameters. Since SCNEC is an aggressive tumor type and requires therapeutic approaches that differ from those used for UC, an accurate diagnosis of SCNEC is critical and this model may help pathologists accurately diagnose SCNEC in daily practice.

**Abstract:**

Although SCNEC is based on its characteristic histology, immunohistochemistry (IHC) is commonly employed to confirm neuroendocrine differentiation (NED). The challenge here is that SCNEC may yield negative results for traditional neuroendocrine markers. To establish an IHC panel for NED, 17 neuronal, basal, and luminal markers were examined on a tissue microarray construct generated from 47 cases of 34 patients with SCNEC as a discovery cohort. A decision tree algorithm was employed to analyze the extent and intensity of immunoreactivity and to develop a diagnostic model. An external cohort of eight cases and transmission electron microscopy (TEM) were used to validate the model. Among the 17 markers, the decision tree diagnostic model selected 3 markers to classify NED with 98.4% accuracy in classification. The extent of synaptophysin (>5%) was selected as the initial parameter, the extent of CD117 (>20%) as the second, and then the intensity of GATA3 (≤1.5, negative or weak immunoreactivity) as the third for NED. The importance of each variable was 0.758, 0.213, and 0.029, respectively. The model was validated by the TEM and using the external cohort. The decision tree model using synaptophysin, CD117, and GATA3 may help confirm NED of traditional marker-negative SCNEC.

## 1. Introduction

Small cell neuroendocrine carcinoma (SCNEC) is a rare entity in the urinary tract, representing 0.5–1% of urinary bladder cancers [1,2]. It usually presents as a high stage tumor with frequent muscularis propria invasion and metastasis compared to conventional urothelial carcinoma (UC) [3]. SCNEC requires an aggressive clinical course, and its 5-year survival rate is as low as 8% [4]. A recently reported combined therapeutic approach included neoadjuvant chemotherapy with cisplatin and etoposide, followed by either radiation therapy or cystectomy if no systemic disease is present; the overall survival was higher in patients who received the neoadjuvant chemotherapy than in those who did not receive it [5,6]. Therefore, accurate diagnosis of SCNEC is critical because of its poor prognosis and therapeutic approaches differing from those used for UC.

SCNEC is defined by its characteristic histology: sheets and large nests of relatively small cells with scant cytoplasm, speckled nuclei, and indistinct nucleoli. In the urinary bladder, SCNEC presents as a pure form or more frequently as a component of combined SCNEC and non-SCNEC [4,7]. The non-SCNEC component includes UC, invasive or in situ, and other divergent differentiation and histologic variants such as squamous, glandular, nested, plasmacytoid, sarcomatoid, and trophoblastic.

The diagnosis of SCNEC is classically based on the histologic features, but immunohistochemical (IHC) staining is commonly employed to confirm the diagnosis or to exclude an alternative diagnosis in cases with ambiguous histology. Similar to its more common counterpart in the lungs, synaptophysin, chromogranin, and CD56 are widely used neuroendocrine (NE) markers in a panel to compensate the suboptimal sensitivity and specificity of each marker [8]. Synaptophysin has a relatively reliable diagnostic potential; chromogranin is less sensitive with weak and focal positivity; and CD56 is most sensitive but less specific [8,9]. However, SCNEC may yield negative results for all three of these markers [10]. In fact, up to two-thirds of small cell lung cancer could provide negative results for the relatively specific NE markers synaptophysin and chromogranin A [10,11]. The challenge is that SCNEC may have ambiguous or overlapping features with UC, especially in cases of combined SCNEC and UC [5]. In such cases, it might be difficult to accurately diagnose SCNEC, and when the traditional NE markers are negative, it could result in misdiagnosis as UC.

Follow-up biopsies are scheduled for bladder cancer patients to estimate treatment response and detect tumor recurrence. While diagnosing a case of SCNEC in the urinary bladder, we found that the previous bladder biopsy had been misdiagnosed as UC because only chromogranin and synaptophysin were tested to define NE differentiation and both tests were negative. This case led us to conduct this present study to define a panel of NE markers to ensure the diagnosis of traditional NE marker-negative SCNEC. We employed a decision tree classifier algorithm to analyze the expression of 17 IHC markers and finally propose a decision tree model using three markers synaptophysin, CD117, and GATA3.

## 2. Materials and Methods

### 2.1. Study Samples

This retrospective study was approved by the Asan Medical Center Institutional Review Board (2013–0107). Initially, the cohort consisted of 47 patients who were diagnosed with SCNEC of the urinary tract (urinary bladder and ureter) as a pure form or combined with UC between May 2002 and October 2020 at Asan Medical Center, Seoul, Republic of Korea. The diagnosis of SCNEC was based on histologic features only or IHC expression analysis of NSE, CD56, chromogranin, and synaptophysin (alone or in combination). After exclusion of 13 patients for which glass slides or paraffin blocks were not available, 34 patients of SCNEC were included in the discovery cohort. Among the 34 patients, 23 patients were biopsied once and accounted for one case each. Nine patients were biopsied twice (accounting for two cases each), and two patients were biopsied thrice (accounting for three cases each). Among the 11 patients who had been biopsied more than once, six patients had specimens diagnosed with UC during the period. The UC cases of these patients were also included in the analysis to compare their immunoprofile with that of SCNEC. Therefore, 34 patients and their 47 cases (40 cases of pure and combined SCNEC and 7 cases of UC) were finally included in the discovery cohort.

For an external validation of the diagnostic model, data for eight patients were retrieved at the Kyung Hee University Medical Center (KHMC), Seoul, Republic of Korea from 2000 to 2020. They had a confirmed or suspected diagnosis of SCNEC of the urinary bladder based on the IHC staining of NE markers.

Patients’ clinicopathological information was obtained from electronic medical records and surgical pathology reports. Pathologic materials of both discovery and external validation cohorts were reassessed according to the 2016 World Health Organization Tumor Classification criteria and staged according to the American Joint Committee on Cancer Staging System, 8th edition.

### 2.2. Tissue Microarray Construction

Tissue microarray blocks with 2-mm-diameter cores were constructed from 10% neutrally buffered formalin-fixed, paraffin-embedded urinary bladder tumor blocks using a tissue microarrayer (Quick-Ray, Unitma Co. Ltd., Seoul, Republic of Korea). In general, three representative cores from each case were generated while trying to exclude necrotic and degenerative areas and to maximize tumor cell content. In cases showing histologically divergent or variant features of UC, each representative area was included, resulting in up to 11 cores generated for one case. As a result, a total of 211 cores were generated.

### 2.3. IHC

IHC analysis was performed using NE, basal, and luminal markers of bladder cancer [11]. The NE markers included in the present study were CD56, CD117, chromogranin, insulinoma-associated protein 1 (INSM1), neuron specific enolase (NSE), SRY (sex determining region Y)-box 2 (SOX2), synaptophysin, somatostatin receptor 2 (SSTR2), and tubulin beta 2B class IIB (TUBB2B). The loss of retinoblastoma-associated protein (Rb) and p53 was reported in bladder cancers with NE differentiation [11,12,13,14]. The basal markers were cytokeratin 5/6 (CK5/6) and cytokeratin 14 (CK14). High expression of epidermal growth factor receptor (EGFR) was reported in the basal subtype of bladder cancer [15]. Luminal markers were cytokeratin 20 (CK20), GATA binding protein 3 (GATA3), and forkhead box A1 (FOXA1) [11,16]. The primary antibodies used in this study, their dilutions, and the subcellular location of each antigen are summarized in Supplementary Table S1. IHC staining was performed using an automated staining system (BenchMark XT, Ventana Medical Systems, Tucson, AZ, USA). The nuclei were counterstained with hematoxylin.

The IHC staining results were assessed in a semiquantitative manner by two pathologists (G.H.K. and S.U.J). The immunoreactivity of the markers was evaluated according to the intensity (negative (0), weak (1), moderate (2), or strong (3)) and the extent of positive tumor cells (percentage). A diffuse expression in a core was defined as immunoreactivity in more than half of tumor cells. The intensity and extent of marker expression were independently assessed in the decision tree analysis.

### 2.4. Establishment of the Decision Tree Model

All 17 IHC markers were included as variables and analyzed for their intensity and extent to classify the cases as neuroendocrine differentiation (NED) and non-neuroendocrine differentiation (non-NED). NED was defined as immunoreactivity to one or more NE markers in cores with SCNEC histology [11]. Based on histologic features and IHC results, the 211 cores were classified into 146 NED cores and 65 non-NED cores. In an attempt to overcome the small number of cases, each core type was analyzed separately to represent NED and non-NED. In cores with simultaneous expression of NE markers with luminal or basal markers, the core was classified as NED when it showed histologic features of SCNEC.

A decision tree model was constructed using a decision tree classifier algorithm on python-3.8, sklearn-1.0.2, and dtreeviz-1.3.2. The algorithm randomly selected 147 cores for the training set and 64 cores for the validation set at odds of 7 to 3. To select a diagnostic IHC panel for NED using the intensity and extent of immunoreactivity of 17 markers, the algorithm repeatedly classified all cores into NED and non-NED to minimize incorrect classifications [17]. A decision tree-derived diagnostic model was visualized after the training procedure was finished. The finally classified cores are colored yellow for NED and green for non-NED in all plots.

### 2.5. Transmission Electron Microscopy (TEM) Analysis

TEM analysis was performed using standard techniques. The submitted tissues were retrieved from paraffin blocks, deparaffinized, post-fixed in 1% buffered osmium tetroxide, dehydrated, and embedded in Epon. Ultrathin sections (1 μm) were stained with uranyl acetate-lead citrate and examined using a JEOL 1200 EX-II TEM (Jeol, Tokyo, Japan) [18].

## 3. Results

### 3.1. Patients’ Characteristics

The clinicopathological features of the 47 cases from the 34 patients are summarized in Table 1. The median age at the initial diagnosis of bladder cancer of the 34 patients was 66 years (range, 31–86 years) with a 6:1 male to female ratio. Most cases were diagnosed by transurethral resection (34 cases, 72.3%) and followed by partial or radical cystectomy (10 cases, 21.3%), ureterectomy (2 cases, 4.3%), and cystoscopic biopsy (1 case, 2.1%). The mean tumor size was 4.36 cm in its greatest dimension (range, 1.0–11.4 cm).

**Figure 1:**
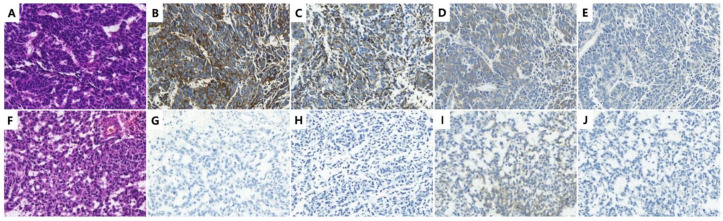
Representative H&E and immunohistochemical images of small cell neuroendocrine carcinoma (SCNEC) of classic histology (**A**–**E**) and with ambiguous histology (**F**–**J**). SCNEC shows sheets of relatively small cells with scant cytoplasm, speckled nuclei, and indistinct nucleoli (**A**). It is typically immunoreactive for synaptophysin (**B**), chromogranin (**C**), and CD117 (**D**) and negative for GATA3 (**E**). SCNEC with ambiguous histology shows sheets of cells with small to medium nuclei, relatively abundant cytoplasm, mild pleomorphism and occasional nucleoli (**F**). Although this case is immunonegative for synaptophysin (**G**) and chromogranin (**H**), the tumor is diffusely immunoreactive for CD117 (**I**) and negative for GATA3 (**J**). (Original magnification: A–I, ×400).

During the reassessment of the cases, we noted that four SCNEC cases from four patients had been misdiagnosed as UC. In three cases, the SCNEC histology was not recognized and IHC for NE markers was not performed. In the remaining case, the SCNEC with ambiguous histology was recognized but chromogranin and synaptophysin staining were negative (Figure 1).

After the reassessment of H&E slides and immune-stained slides, the cases were classified as pure SCNEC (29 cases, 61.7%), combined SCNEC and UC (15 cases, 31.9%), and UC (3 cases, 6.4%). Divergent differentiation and variant histology were frequently noted and included glandular (6 cases, 12.7%) and squamous (3 cases, 6.4%) differentiation and micropapillary (4 cases, 8.5%), rhabdoid (1 case, 2.1%), and giant cell (1 case, 2.1%) variants. Tumor invasion into the muscularis propria was noted in 38 cases (80.9%). Twenty-five patients were treated with chemotherapy. Among the 10 cases involving partial or radical cystectomy, most were of high pathologic stages with pT3 (8 cases, 80%) and pT4 (1 case, 10%), and half of the patients had lymph node metastasis (5 patients, 50.0%).

### 3.2. Expression of NE, Luminal, and Basal Markers in the Discovery Cohort

The expression profile of 17 IHC markers in the 146 NED cores and 65 non-NED cores is summarized in Table 2. Detailed information on the IHC markers is presented in Supplementary Table S2. Representative IHC images are presented in Supplementary Figure S1.

In the NED cores, synaptophysin was the most strongly and widely expressed NE marker, and approximately 80% of NED cores showed diffuse expression. CD56 and CD117 were also diffusely expressed in 61.0% and 58.2% of NED cores, respectively. However, a subset of NED cores was negative for the NE markers synaptophysin (12 cores, 8.2%), CD56 (30 cores, 20.5%), and CD117 (38 cores, 26.0%). Chromogranin and INSM1were expressed less widely, and their diffuse expression was noted in 20.5% and 43.8% of NED cores, respectively. As expected, the expression of luminal (CK20 and GATA3) and basal (CK5/6 and CK14) markers was negative or weak in ≤5% NED cores. However, EGFR and FOXA1 were expressed in a significant number of NED cores and immunoreactive in 31.5% and 71.9% of NED cores, respectively, with varying intensities.

In the non-NED cores, most of the NE markers such as synaptophysin, chromogranin, CD56, INSM1, SSTR2, and CD117 were negative or weakly expressed (≤5%) in more than 95% of such cores. NSE, SOX2, and TUBB2 were immunoreactive in a significant extent (>5%) of non-NED cores (43.0%, 44.6%, and 13.8%, respectively) with varying intensities, although they were expressed as such in most NED cores (86.3%, 79.5%, 53.4%, respectively). GATA3 and EGFR showed diffuse expression in 80.0% and 73.9% of non-NED cores, respectively.

### 3.3. Decision Tree-Based Diagnostic NE IHC Model

Given the lack of expression of NE markers in a significant number of NED cores, the decision tree classifier algorithm was employed to define a diagnostic IHC panel for NED. Among multiple models suggested by the algorithm, this model was selected because it was relatively simple, highly reproducible, and easy to apply in routine clinical practice. It consisted of three markers synaptophysin (cutoff >5% immunoreactive area), CD117 (cutoff >20% immunoreactive area), and GATA3 (cutoff of negative/weak intensity to be classified as NED) and applied in that order. The relative importance of the markers was 0.758 for synaptophysin, 0.213 for CD117, and 0.029 for GATA3 in the model.

**Figure 2:**
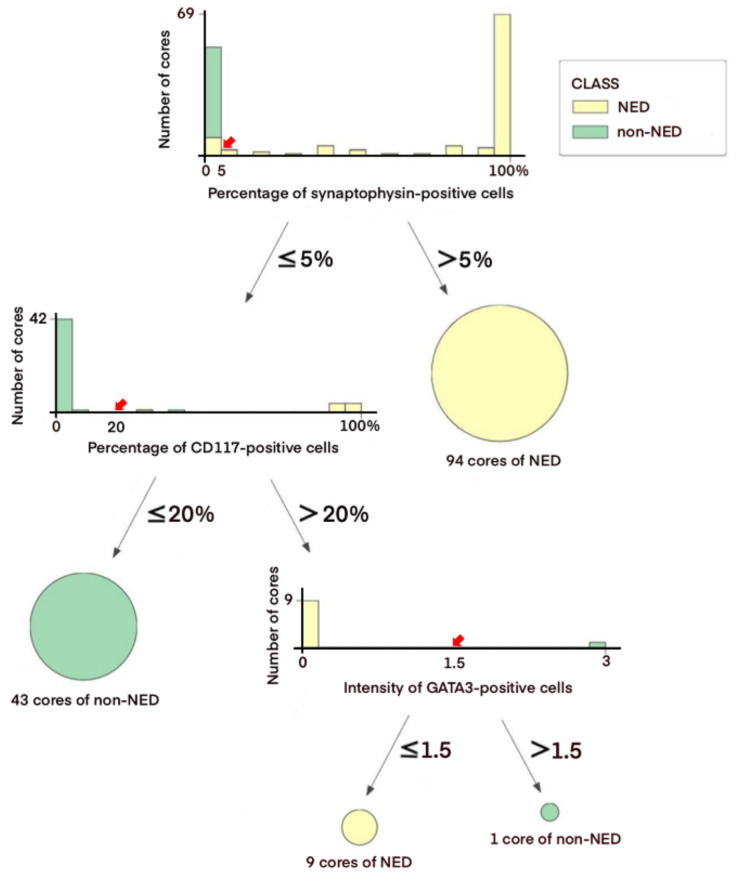
Decision tree model of the discovery cohort. Diagnostic flow of the training set is demonstrated with cutoff values (bold red arrow) and distribution plots of NED and non-NED cores. Each distribution plot stands for a split-by-condition node. The *x*-axis and *y*-axis represent the extent or intensity of the corresponding IHC marker and the number of NED or non-NED cores, respectively. The finally classified cores are colored yellow for NED and green for non-NED. The degrees of intensity of GATA3 are represented as follows: 0, negative; 1, weak; 2, moderate; 3, strong.

An overview of the decision tree model using 147 cores of the training set is shown in Figure 2. The synaptophysin immunoreactivity was noted in >5% tumor area in 94 cores and was classified as NED (64.0%). Among 53 cores with ≤5% synaptophysin-immunoreactive area, 43 cores were of CD117-immunoreactive area ≤20% and classified as non-NED (81.1%). In cores with the CD117-immunoreactive area >20%, the intensity of GATA3 immunoreactivity was considered, being classified as NED in 9 cores with negative/weak intensity (90.0%) and non-NED in 1 core with moderate to strong intensity (10.0%) (Supplementary Figure S2). The overall accuracy and area under the receiver operating characteristic curve were 98.4% and 98.8% according to the internal validation.

**Figure 3:**
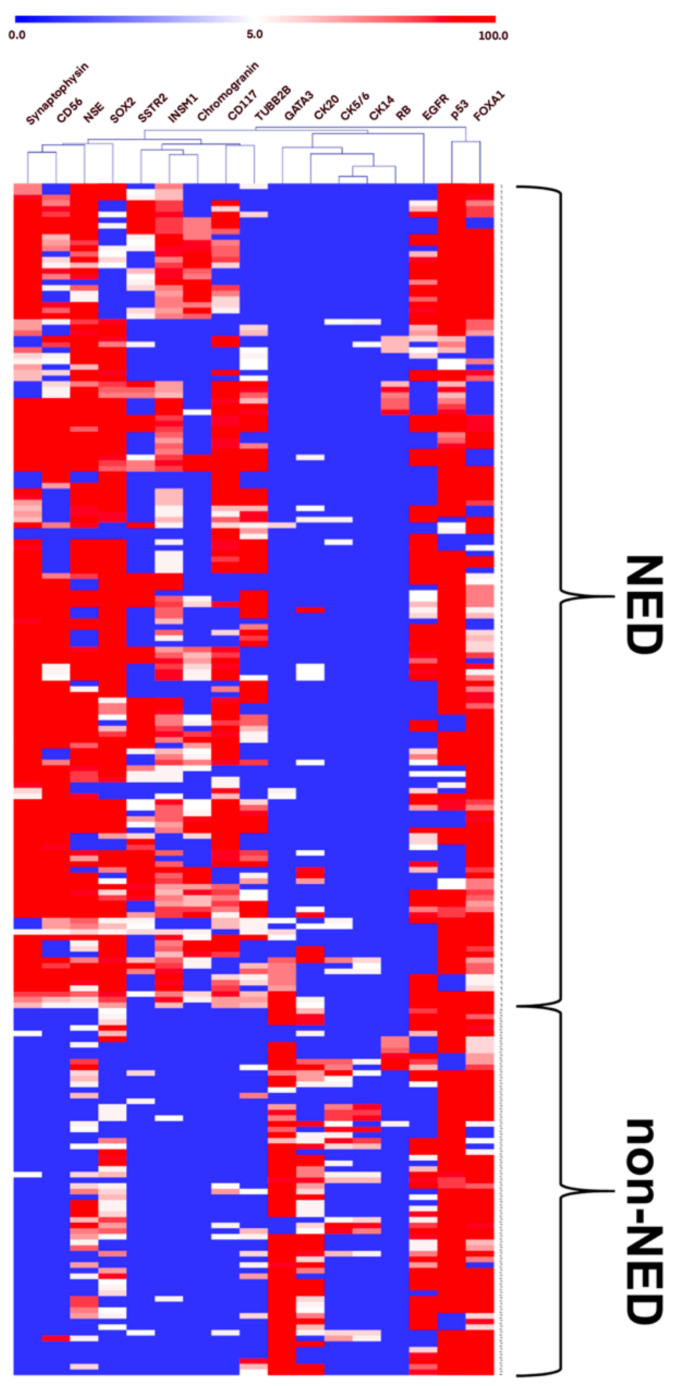
Distribution of the expression of 17 markers in NED and non-NED cores. Heatmap of 17 markers is presented. The white to red shades show increasing immunoreactivity from 5% to 100%, and the blue color represents less than 5% immunoreactivity of IHC markers including no expression. See color scale.

The distribution of expression and association of each marker in all cores of the discovery cohort are presented in Figure 3. When the decision tree model was applied to all 211 cores, 11 cores with ≤5% of synaptophysin-immunoreactive area were classified as NED. They expressed one or more NE markers such as CD117 (11/11 cores, 100%), CD56 (9/11 cores; 81.8%), TUBB2B (6/11 cores, 54.6%), SOX2 (9/11 cores, 81.8%), NSE (7/11 cores, 63.6%), SSTR2 (5/11 cores, 45.5%), and INSM1 (3/11 cores, 27.3%). According to the model, CD117 expression was identified in all NED cores with ≤5% of synaptophysin-immunoreactive area and showed a weak relationship with synaptophysin compared to other NE markers.

### 3.4. Application of the Diagnostic NE IHC Model on an External Cohort

Six SCNEC cases and two UC cases from the external cohort were immunostained for synaptophysin, CD117, and GATA3 using whole tumor sections in our institution. According to the model, five SCNEC cases were immunoreactive for synaptophysin in more than 20% of tumor cells and classified as NED. The remaining SCNEC case was negative for synaptophysin but immunoreactive for CD117 in more than 90% of tumor cells, being classified as NED. The two UC cases were immunonegative for all three markers and classified as non-NED. These results were consistent with the original diagnosis.

### 3.5. Ultrastructural Validation of NE Differentiation

TEM was performed on samples from five SCNEC cases (four cases in the discovery cohort from which the 11 cores with ≤ 5% of synaptophysin-immunoreactive area were derived and one such case from the external cohort). Two SCNEC cases with diffuse synaptophysin expression and two UC cases were also included as positive and negative controls, respectively.

**Figure 4:**
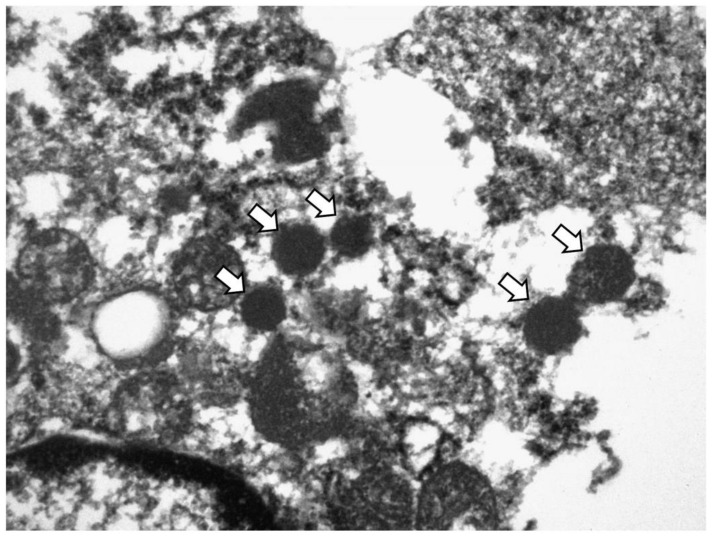
Transmission electron microscopy image of synaptophysin-negative SCNEC. Arrows indicate neurosecretory granules (218.31–275.16 nm). (Original magnification, ×20,000).

All five cases showed varied numbers of electron dense neurosecretory granules in the cytoplasm of the tumor cells, similar to those of SCNEC (Figure 4). They ranged from 144.5 to 582.2 nm. The granules were round with a dense core, although the delimiting outer membrane and peripheral halos were not clearly observed probably due to the deparaffinization process. There were no neurosecretory granules in the two UC cases (data not shown).

## 4. Discussion

Herein, we propose a decision tree-based IHC model consisting of two inclusion markers synaptophysin and CD117 and one exclusion marker GATA3 for the diagnosis of SCNEC of the urinary bladder. It could detect NED of not only NE marker-positive SCNEC but also traditional marker-negative SCNEC. The model was validated using an external cohort and by TEM analysis.

Through this study, we emphasize the following points for the diagnosis of SCNEC. First, it is crucial to be familiar with the histological features of SCNEC. In cases with ambiguous histological features that are difficult to differentiate from UC, IHC for NE markers should be performed with a low threshold. Second, even focal (>5%) and weak synaptophysin immunoreactivity would be sufficient for the diagnosis of SCNEC. Third, in synaptophysin-negative cases, CD117 and GATA3 may be helpful to distinguish between SCNEC and non-SCNEC.

SCNEC is mainly diagnosed based on histology and may not require IHC confirmation. As reported previously, most of our cases including traditional NE marker-negative cases showed classic histological features of SCNEC. The tumor presented as solid sheets, nests, or trabeculae of small cells. Tumor cells have sparse cytoplasm, nuclear molding, finely granular stippled chromatin, inconspicuous nucleoli, high mitotic count, and frequent individual and geographic necrosis [4]. However, ambiguous histological features such as relatively abundant cytoplasm and the presence of nucleoli albeit inconspicuous were noted as shown in Figure 1. In such cases, IHC for NE markers might be useful to confirm NED.

Synaptophysin, chromogranin, and CD56 are widely used clinically in a diagnostic panel because of their suboptimal sensitivity and specificity as individual markers [9]. In the more common counterpart lung cancer, synaptophysin is expressed in 41–75% of small cell lung carcinoma (SCLC) and 58–85% of large cell neuroendocrine carcinomas (LCNEC). Chromogranin may show weak and focal positivity and less sensitivity, being expressed in only 23–58% of SCLC and 42–69% of LCNEC. CD56 is expressed in most SCLC (72–99%) and LCNEC (72–94%) cases but at the cost of relatively low specificity (72%). As expected synaptophysin was chosen as the most important NE marker in our model.

CD117 was chosen as the second most important marker for the diagnosis of SCNEC in preference to other traditional or emerging NE markers. This could be explained, at least in part, by the fact that other NE markers were often expressed simultaneously whereas CD117 was expressed in those NE marker-negative SCNEC cases. CD117 expression has been reported in SCNEC of various organs such as the lung, uterine cervix, and esophagus [19,20,21]. CD117 expression was also noted in 27% cases of SCNEC in the urinary bladder [22]. The mechanisms of CD117 expression in NE carcinoma are largely unknown, but an autocrine growth loop has been suggested in SCLC cell lines [23]. As a member of the type III receptor tyrosine kinase family, CD117 activates several signaling pathways, such as the JAK/STAT, RAS/MAP kinase pathway, PI3 kinase, PLCγ pathway, and SRC pathway [24]. Consequently, it plays an important role in the proliferation, survival, differentiation, apoptosis, and migration of tumor cells [24]. Another hypothesis is that CD117 may increase cancer stem cell phenotype in SCNEC since it plays a key role in maintaining the stemness of cancer stem cells [24]. Because both UC and SCNEC arise from common multipotential cancer stem cells, SCNEC frequently coexists with conventional UC [25]. Therefore, CD117 expression may represent a marker of aggressive biologic behavior of SCNEC instead of NED in the model.

According to previous reports, a novel pan-NE marker INSM1 was superior to traditional NE markers with high sensitivity (93.9%) and specificity (97.4%) in the SCNEC of the genitourinary tract [26,27]. In our cases, INSM1 showed relatively lower sensitivity (78.1%) but similar high specificity (96.9%) compared to the previous report. Nevertheless, this novel marker was not selected in our model. The decision tree model suggests variables based on the causal relationship and selects the best one if multiple variables are correlated. As shown in Figure 3, when there is a strong relationship between INSM1 and synaptophysin immunoreactivity, synaptophysin might be selected in the model.

Among non-NE markers employed in the present study, GATA3 immunoreactivity was selected as an exclusion marker for NE differentiation probably because of its relatively higher specificity than that of the other non-NE markers. The basal markers CK5/6 and CK14 were not only negative in most NE cores (94.5% and 93.8%, respectively) but also not expressed in more than half of non-NE cores (63.1% and 66.2%, respectively). The luminal marker FOXA1 was expressed similarly in NE cores and non-NE cores (88.4% and 83.1%, respectively). In the remaining luminal markers, GATA3 was negative in more NE cores than CK20 (89.7% and 81.5%, respectively) and had stronger immunoreactivity in the non-NE cores (moderate to strong immunoreactivity in 89.3% and 75.3%, respectively). Therefore, basal markers CK5/6 and CK14 and luminal marker FOXA1 might offer suboptimal distinguishing power between NE cores and non-NE cores, and GATA3 might be a better exclusion marker than CK20.

Although the demand for TEM has decreased due to the development of IHC staining and molecular pathology, this technique is still used for accurate diagnosis. TEM is particularly useful for the differential diagnosis between malignant mesothelioma and serous carcinoma, whereas immunostaining results alone cannot achieve an accurate diagnosis [28]. In the present study, neurosecretory granules were found in all synaptophysin-negative and inconspicuous (≤5%) cases and were useful for confirming NED in those cases, although the number of granules was fewer than that in classic SCNEC cases.

Genomic analyses of bladder cancer have been used for the molecular characterization of variant histologic subtypes. The Cancer Genome Atlas (TCGA) and a report by Lund et al. have identified neuronal subtype or small cell/neuroendocrine (SC/NE) consensus cluster, accounting for 3–15% of bladder cancer by RNA-sequencing analysis [16,29,30]. A TCGA report has shown that tumors representing NED at the molecular level were not similar in histology to SCNEC in 85% of cases (17/20) [16]. A report by Lund et al. showed that only half of the SC/NE consensus cluster represented the enriched expression of neuronal markers such as synaptophysin, chromogranin, and CD56 [29]. Phenotypical UC with the absence of NE histology may also reveal transcriptomic patterns of NE carcinoma and be defined as neuroendocrine-like (NE-like) tumors [11]. These reports suggest that histological, molecular, and IHC results of SCNEC may not agree completely with each other. Combining our findings with previous results, continuous efforts should be made to define the diagnostic criteria for aggressive NE carcinoma that requires therapeutic approaches different from those used for UC.

The present study has limitations. Although the performance of the decision tree diagnostic model was excellent, the possibility of overfitting cannot be excluded. Since we performed core-based analysis to compensate for the small number of SCNEC cases, this model needs to be validated with larger numbers of SCNEC cases, preferably in a multicenter study.

## 5. Conclusions

Our study demonstrated that the decision tree model using synaptophysin, CD117, and GATA3 may help confirm NED of not only NE marker-positive SCNEC but also traditional marker-negative SCNEC.

## Figures and Tables

**Table 1:** Clinicopathological features of the discovery cohort.

Features		Value
Patients (*n* = 34)		
Age at initial diagnosis (years)		66.1 (31–86)
Sex	Male	29 (85.3)
	Female	5 (14.7)
All cases (*n* = 47)		
Tumor size (cm)		4.36 (1.0–11.4)
Location	Urinary bladder	45 (95.7)
	Ureter	2 (4.3)
Procedure	Cystoscopic biopsy	1 (2.1)
	Transurethral resection	34 (72.3)
	Partial cystectomy	2 (4.3)
	Radical cystectomy/ureterectomy	10 (21.3)
Histology	Pure NEC	29 (61.7)
	Mixed NEC and non-NEC	15 (31.9)
	Non-NEC	3 (6.4)
Invasion depth	Non-invasive	0 (0.0)
	Subepithelial connective tissue	9 (19.1)
	Muscularis propria	28 (59.6)
	Perivesical tissue	9 (19.1)
	Other organs *	1 (2.1)
Lymphovascular invasion	Present	25 (53.2)
	Absent	22 (46.8)
Cystectomy cases (*n* = 10)		
Tumor stage	pT1	0 (0.0)
	pT2	1 (10.0)
	pT3	8 (80.0)
	pT4	1 (10.0)
N stage	NX	1 (10.0)
	N0	4 (40.0)
	N1-3	5 (50.0)

* Other organs: prostate, both seminal vesicles, and right vas deferens.

**Table 2:** Immunoprofile of neuroendocrine cores and non-neuroendocrine cores from small cell neuroendocrine carcinomas of the urinary tract.

	Neuroendocrine Cores (*n* = 146)										Non-Neuroendocrine Cores (*n* = 65)										
	Intensity				Extent						Intensity				Extent						
	0 and 1		2 and 3		≤5%		>5–≤50%		>50%		0 and 1		2 and 3		≤5%		>5–≤50%		>50%		
SYP	29	(19.9)	117	(80.1)	12	(8.2)	18	(12.3)	116	(79.5)	65	(100)	0	(0.0)	65	(100)	0	(0.0)	0	(0.0)	
CGA	84	(57.5)	62	(42.5)	89	(61.0)	27	(18.5)	30	(20.5)	65	(100)	0	(0.0)	65	(100)	0	(0.0)	0	(0.0)	
CD56	47	(32.2)	99	(67.8)	32	(21.9)	25	(17.1)	89	(61.0)	63	(96.9)	2	(3.1)	64	(98.5)	0	(0.0)	1	(1.5)	
CD117	74	(50.7)	72	(49.3)	38	(26.0)	23	(15.8)	85	(58.2)	61	(93.8)	4	(6.2)	62	(95.4)	3	(4.6)	0	(0.0)	
INSM1	43	(29.5)	103	(70.5)	33	(22.6)	49	(33.6)	64	(43.8)	65	(100)	0	(0.0)	64	(98.5)	1	(1.5)	0	(0.0)	
NSE	35	(24.0)	111	(76.0)	20	(13.7)	15	(10.3)	111	(76.0)	45	(69.2)	20	(30.8)	37	(56.9)	19	(29.2)	9	(13.8)	
SOX2	25	(17.1)	121	(82.9)	30	(20.5)	16	(11.0)	100	(68.5)	27	(41.5)	38	(58.5)	36	(55.4)	21	(32.3)	8	(12.3)	
TUBB2B	78	(53.4)	68	(46.6)	68	(46.6)	27	(18.5)	51	(34.9)	54	(83.1)	11	(16.9)	56	(86.2)	8	(12.3)	1	(1.5)	
SSTR2	78	(53.4)	68	(46.6)	81	(55.5)	23	(15.8)	42	(28.8)	62	(95.4)	3	(4.6)	63	(96.9)	2	(3.1)	0	(0.0)	
p53	17	(11.6)	129	(88.4)	26	(17.8)	9	(6.2)	111	(76.0)	15	(23.1)	50	(76.9)	9	(13.8)	0	(0.0)	56	(86.2)	
Rb	131	(89.7)	15	(10.3)	130	(89.0)	8	(5.5)	8	(5.5)	65	(100)	0	(0.0)	65	(100)	0	(0.0)	0	(0.0)	
EGFR	95	(65.1)	51	(34.9)	81	(55.5)	19	(13.0)	46	(31.5)	10	(15.4)	55	(84.6)	6	(9.2)	11	(16.9)	48	(73.8)	
CK5/6	138	(94.5)	8	(5.5)	142	(97.3)	4	(2.7)	0	(0.0)	41	(63.1)	24	(36.9)	46	(70.8)	11	(16.9)	8	(12.3)	
CK14	137	(93.8)	9	(6.2)	143	(97.9)	3	(2.1)	0	(0.0)	43	(66.2)	22	(33.8)	50	(76.9)	9	(13.8)	6	(9.2)	
CK20	119	(81.5)	27	(18.5)	135	(92.5)	4	(2.7)	7	(4.8)	17	(26.2)	48	(73.8)	21	(32.3)	22	(33.8)	22	(33.8)	
FOXA1	39	(26.7)	107	(73.3)	18	(12.3)	23	(15.8)	105	(71.9)	23	(35.4)	42	(64.6)	14	(21.5)	15	(23.1)	36	(55.4)	
GATA3	131	(89.7)	15	(10.3)	134	(91.8)	8	(5.5)	4	(2.7)	8	(12.3)	57	(87.7)	9	(13.8)	4	(6.2)	52	(80.0)	

Data are expressed as number (%). Abbreviations: SYP, synaptophysin; CGA, chromogranin; INSM1, insulinoma-associated protein 1; NSE, neuron specific enolase; SOX2, SRY (sex determining region Y)-box 2; TUBB2B, tubulin beta 2B class IIb, SSTR2, somatostatin receptor 2; p53, tumor protein p53; Rb, retinoblastoma-associated protein; EGFR, epidermal growth factor receptor; CK5/6, cytokeratin 5/6; CK14, cytokeratin 14; CK20, cytokeratin 20; FOXA1, forkhead box A1; GATA3, GATA binding protein 3.

## Data Availability

The data are available on request from the corresponding author.

## Supplementary Materials

The following supporting information can be downloaded at: https://www.mdpi.com/article/10.3390/cancers14102495/s1, Figure S1: Representative immunohistochemical analysis of 17 markers used in the present study.; Figure S2: Representative immunohistochemistry of GATA3. Table S1: Antibodies used in the study.; Table S2: Immunoprofile of neuroendocrine cores and non-neuroendocrine cores from small cell neuroendocrine carcinomas of the urinary tract.
