# Supplementary material for: Synaptophysin, CD117, and GATA3 as a Diagnostic Immunohistochemical Panel for Small Cell Neuroendocrine Carcinoma of the Urinary Tract
Source: Cancers (Basel). 2022 May 19;14(10):2495. doi: 10.3390/cancers14102495 (PMC9139575; doi:10.3390/cancers14102495)
Supplement: Supplementary file 1 [file cancers-14-02495-s001.zip › cancers-1714891-Supplementary.pdf]

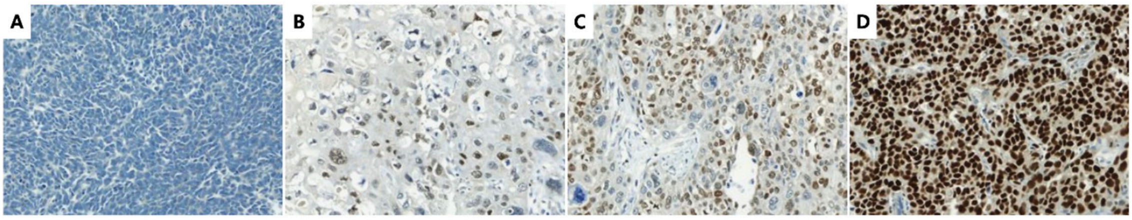

Figure S1. Representative immunohistochemical analysis of 17 markers used in the present study.

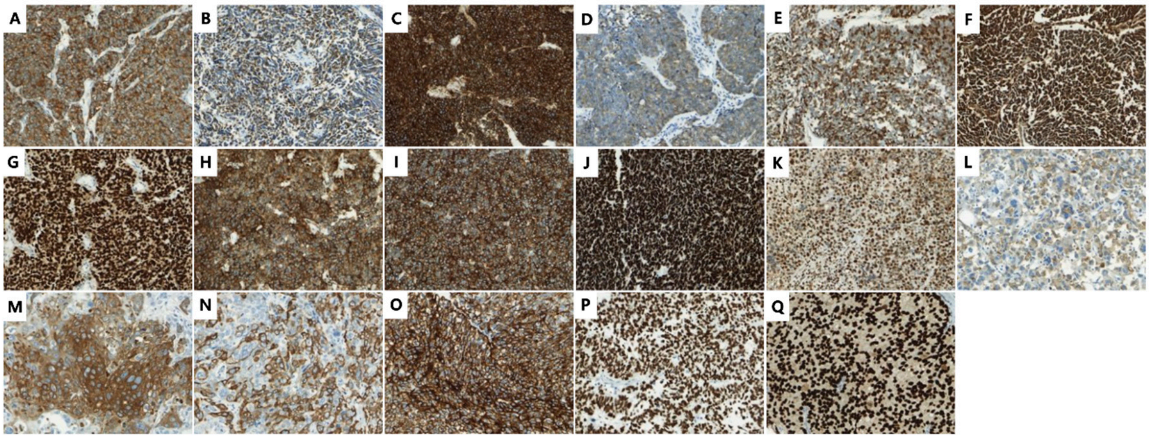

Figure S2. Representative immunohistochemistry of GATA3.

Supplementary Table S1. Antibodies used in the study.

Antibody	Clone	Supplier	Dilution	Subcellular location
Neuroendocrine markers				
Synaptophysin	336R-96	Cell Marque	1:200	Cell membrane, cytoplasm
Chromogranin	M0869	DAKO	1:1600	Cell membrane, cytoplasm
CD56	NCL-L-CD56-504	NOVO	1:100	Cell membrane, cytoplasm
CD117	117R-16	Cell Marque	1:200	Cell membrane, cytoplasm
INSM1	SC-271408	Santacruz	1:100	Nucleus
NSE	M0873	DAKO	1:400	Cell membrane, cytoplasm
SOX2	Ab92494	Abcam	1:50	Nucleus
TUBB2B	LS-B4190	LSbio	1:1000	Cell membrane, cytoplasm
SSTR2	Ab134152	Abcam	1:6400	Cell membrane, cytoplasm
p53	M7001	DAKO	1:1000	Nucleus
Rb	#3107	QED Bioscience	1:10000	Nucleus
EGFR	414R-16	Cell Marque	1:100	Cell membrane, cytoplasm
Basal markers				
CK5/6	M7237	DAKO	1:200	Cell membrane, cytoplasm
CK14	314M-14	Cell Marque	1:200	Cell membrane, cytoplasm
Luminal markers				
CK20	M7019	DAKO	1:200	Cell membrane, cytoplasm
FOXA1	SC-101058	Santacruz	1:100	Nucleus
GATA3	390M-16	Cell Marque	1:100	Nucleus

Supplementary Table S2. Immunoprofile of neuroendocrine cores and non-neuroendocrine cores from small cell neuroendocrine carcinomas of the urinary tract.

	NECUB (n=146)																	
	Intensity (%)								Extent (%)									
Antibody	0		1		2		3		≤ 5		6-20		21-50		51-80		> 80	
Synaptophysin	12	(8.2)	17	(11.6)	36	(24.7)	81	(55.5)	12	(8.2)	5	(3.4)	13	(8.9)	8	(5.5)	108	(74.0)
Chromogranin	82	(56.2)	2	(1.4)	15	(10.3)	47	(32.2)	89	(61.0)	14	(9.6)	13	(8.9)	10	(6.8)	20	(13.7)
CD56	30	(20.5)	17	(11.6)	28	(19.2)	71	(48.6)	32	(21.9)	13	(8.9)	12	(8.2)	10	(6.8)	79	(54.1)
CD117	38	(26.0)	36	(24.7)	52	(35.6)	20	(13.7)	38	(26.0)	12	(8.2)	11	(7.5)	32	(21.9)	53	(36.3)
INSM1	32	(21.9)	11	(7.5)	35	(24.0)	68	(46.6)	33	(22.6)	13	(8.9)	36	(24.7)	50	(34.2)	14	(9.6)
NSE	20	(13.7)	15	(10.3)	14	(9.6)	97	(66.4)	20	(13.7)	9	(6.2)	6	(4.1)	22	(15.1)	89	(61.0)
SOX2	23	(15.8)	2	(1.4)	5	(3.4)	116	(79.5)	30	(20.5)	7	(4.8)	9	(6.2)	16	(11.0)	84	(57.5)
TUBB2B	61	(41.8)	17	(11.6)	44	(30.1)	24	(16.4)	68	(46.6)	15	(10.3)	12	(8.2)	14	(9.6)	37	(25.3)
SSTR2	69	(47.3)	9	(6.2)	16	(11.0)	52	(35.6)	81	(55.5)	14	(9.6)	9	(6.2)	4	(2.7)	38	(26.0)
P53	16	(11.0)	1	(0.7)	0	(0.0)	129	(88.4)	26	(17.8)	2	(1.4)	7	(4.8)	5	(3.4)	106	(72.6)
Rb	130	(89.0)	1	(0.7)	7	(4.8)	8	(5.5)	130	(89.0)	1	(0.7)	7	(4.8)	6	(4.1)	2	(1.4)
EGFR	74	(50.7)	21	(14.3)	34	(23.3)	17	(11.6)	81	(55.5)	11	(7.5)	8	(5.5)	8	(5.5)	38	(26.0)
CK5/6	138	(94.5)	0	(0.0)	1	(0.7)	7	(4.8)	142	(97.3)	3	(2.0)	1	(0.7)	0	(0.0)	0	(0.0)
CK14	137	(93.8)	0	(0.0)	0	(0.0)	9	(6.2)	143	(98.0)	3	(2.0)	0	(0.0)	0	(0.0)	0	(0.0)
CK20	119	(81.5)	0	(0.0)	7	(4.8)	20	(13.7)	135	(92.5)	3	(2.0)	1	(0.7)	2	(1.4)	5	(3.4)
FOXA1	17	(11.6)	22	(15.1)	9	(6.2)	98	(67.1)	18	(12.3)	8	(5.5)	15	(10.3)	21	(14.4)	84	(57.5)
GATA3	131	(89.7)	0	(0.0)	7	(4.8)	8	(5.5)	134	(91.8)	3	(2.0)	5	(3.4)	1	(0.7)	3	(2.0)

	Non-NECUB (n=65)																	
	Intensity (%)								Extent (%)									
Antibody	0		1		2		3		≤ 5		6-20		21-50		51-80		> 80	
Synaptophysin	63	(96.9)	2	(3.1)	0	(0.0)	0	(0.0)	65	(100.0)	0	(0.0)	0	(0.0)	0	(0.0)	0	(0.0)
Chromogranin	65	(100.0)	0	(0.0)	0	(0.0)	0	(0.0)	65	(100.0)	0	(0.0)	0	(0.0)	0	(0.0)	0	(0.0)
CD56	63	(96.9)	0	(0.0)	2	(3.1)	0	(0.0)	64	(98.5)	0	(0.0)	0	(0.0)	1	(1.5)	0	(0.0)
CD117	60	(92.3)	1	(1.5)	2	(3.1)	2	(3.1)	62	(95.4)	2	(3.1)	1	(1.5)	0	(0.0)	0	(0.0)
INSM1	63	(96.9)	2	(3.1)	0	(0.0)	0	(0.0)	64	(98.5)	1	(1.5)	0	(0.0)	0	(0.0)	0	(0.0)
NSE	34	(52.3)	11	(16.9)	16	(24.6)	4	(6.2)	37	(56.9)	10	(15.4)	9	(13.8)	4	(6.2)	5	(7.7)
SOX2	27	(41.5)	0	(0.0)	10	(15.4)	28	(43.1)	36	(55.4)	14	(21.5)	7	(10.8)	4	(6.2)	4	(6.2)
TUBB2B	53	(81.5)	1	(1.5)	8	(12.3)	3	(4.6)	56	(86.2)	4	(6.2)	4	(6.2)	1	(1.5)	0	(0.0)
SSTR2	61	(93.8)	1	(1.5)	3	(4.6)	0	(0.0)	63	(96.9)	2	(3.1)	0	(0.0)	0	(0.0)	0	(0.0)
P53	6	(9.2)	9	(13.8)	0	(0.0)	50	(76.9)	9	(13.8)	0	(0.0)	0	(0.0)	7	(10.8)	49	(75.4)
Rb	65	(100.0)	0	(0.0)	0	(0.0)	0	(0.0)	65	(100.0)	0	(0.0)	0	(0.0)	0	(0.0)	0	(0.0)
EGFR	4	(6.2)	6	(9.2)	22	(33.8)	33	(50.8)	6	(9.2)	4	(6.2)	7	(10.8)	9	(13.8)	39	(60.0)
CK5/6	41	(63.1)	0	(0.0)	4	(6.2)	20	(30.8)	46	(70.8)	6	(9.2)	5	(7.7)	6	(9.2)	2	(3.1)
CK14	43	(66.2)	0	(0.0)	1	(1.5)	21	(32.3)	50	(76.9)	6	(9.2	3	(4.6)	6	(9.2	0	(0.0)
CK20	16	(24.6)	1	(1.5)	14	(21.5)	34	(52.3)	21	(32.3)	13	(20.0)	9	(13.8)	7	(10.8)	15	(23.1)
FOXA1	11	(16.9)	12	(18.5)	7	(10.8)	35	(53.8)	14	(21.5)	8	(12.3)	7	(10.8)	9	(13.8)	27	(41.5)
GATA3	8	(12.3)	0	(0.0)	2	(3.1)	55	(84.6)	9	(13.8)	0	(0.0)	4	(6.2)	4	(6.2)	48	(73.8)
